# The effects of benralizumab on lung volume changes during exercise in experimental setting in severe asthmatics: a pilot study

**DOI:** 10.3389/fphar.2025.1611168

**Published:** 2025-07-23

**Authors:** Alida Benfante, Alessandra Tomasello, Lorena Gentile, Alessia Leonarda Lisotta, Paola Marasà, Salvatore Battaglia, Nicola Scichilone

**Affiliations:** Department of Health Promotion, Mother and Child Care, Internal Medicine and Medical Specialties (ProMISE), University of Palermo, Palermo, Italy

**Keywords:** dynamic hyperinflation, inspiratory capacity, severe asthma, exercise, benralizumab

## Abstract

**Introduction:**

The most severe forms of asthma are characterised by the occurrence of dyspnoea on exertion, impacting on daily activities and quality of life. It has been demonstrated that dynamic hyperinflation (DH) during exercise represents a mechanism of physical activity limitation in severe asthmatics. Inspiratory capacity (IC) at rest may be an indicator of static hyperinflation, and the change in IC during exercise can be regarded as a marker of DH. The study aims to assess whether Benralizumab is able to improve IC and to reduce DH.

**Materials and methods:**

A pilot study on severe asthmatics was conducted. Assessments of asthma control and quality of life, lung function evaluation and 6-min walk test (6MWT) were carried out on the day of the first drug administration (T_0_) and after a period of 6 months (T_1_).

**Results:**

Twelve severe asthmatics were recruited. Significant improvements of dynamic volumes, asthma control and quality of life were observed after anti IL-5R treatment. At T_0_, pre-6MWT-IC and post-6MWT-IC were 2.40 ± 0.48 L and 1.60 ± 0.83 L, respectively (p < 0.0001). Minute ventilation (VE) at the beginning of the 6MWT was 13.88 ± 4.44 L·min^−1^ and post 6MWT was 23.77 ± 12.11 L·min^−1^ (p < 0.0001). At T_1_, IC pre 6MWT was higher than IC pre 6MWT at T_0_ (2.74 ± 1.14, p = 0.010) and did not change after exercise (IC post 6MWT: 2.85 ± 1.22 L, p = 0.53). VE did not change at T_1_.

**Discussion:**

These findings show the effect of Benralizumab in improving IC during exercise. The disappearance of DH provides a potential explanation for the beneficial effect of biologics in severe asthmatics.

## Introduction

Asthma is a widespread disease throughout the world, which hinders the performance of daily activities and reduces the quality of life of affected patients, especially in the most severe forms (Chung et al., 2014; Wilson et al., 2012). The most common and problematic symptom among asthmatic patients is exertional dyspnea, which has been hypothesized to be associated with the occurrence of dynamic hyperinflation (DH) (Haselkorn et al., 2010). DH is a well-recognized cause of physical exercise limitation among COPD patients, and is sustained by reduced alveolar attachments and consequent impaired lung elastic recoil; on the other hand, its occurrence in asthma is less investigated (Vermeulen et al., 2016), and could be attributed to different causes. In a recent study by van der Meer and colleagues (van der Meer et al., 2021), DH was shown to be associated with features of airway inflammation in moderate to severe asthmatics, and to decrease with systemic corticosteroids, thus being recognized as an important treatable trait. The same authors previously demonstrated that DH is associated with impaired physical activity in severe asthmatics (van der Meer et al., 2019).

Patients affected by severe asthma may require biologic drugs to achieve control of respiratory symptoms. Benralizumab is a monoclonal antibody directed against the IL-5 receptor, which has been proved safe and effective in reducing the number of disease exacerbations, hospitalizations and the dose of oral corticosteroids in patients affected by severe eosinophilic asthma (Nair et al., 2017; Bleecker et al., 2016; FitzGerald et al., 2016). Recent studies (Pelaia et al., 2020; Panettieri et al., 2020; Maniscalco et al., 2024) have shown a tendency to improvement in static lung volumes after treatment with benralizumab, which could further explain the clinical effects on daily activities.

In this pilot study, we sought to demonstrate that anti-IL5 receptor α monoclonal antibody is effective in reducing lung hyperinflation in severe asthmatics. For this purpose, we assessed the changes in inspiratory capacity (IC) in severe asthmatic subjects measured during the 6-min walking test (6MWT), a condition resembling daily physical activities in experimental setting.

## Materials and methods

### Study population

The study was conducted at the PROMISE Department of the University Hospital of Palermo, Italy. Patients aged between 18 and 65 years suffering from severe eosinophilic asthma according to GINA guidelines (Global Initiative for Asthma, 2024), who were eligible for treatment with an anti-IL-5R monoclonal antibody were recruited. The eligibility criteria (at least 2 out of 3 required) are: a) eosinophil value ≥ 300/mmc in the absence of systemic steroid treatment; b) at least two asthma exacerbations despite maximum inhaled therapy (Step 4–5 GINA) treated with systemic steroid or requiring hospitalization in the previous 12 months; c) continuous therapy with oral steroids, in addition to maximum inhaled therapy in the last year. Patients who smoked or suffered from respiratory diseases other than bronchial asthma were excluded from the study. All subjects were under inhaled corticosteroids/beta-2 adrenergic long-acting bronchodilators (ICS/LABA) therapy in a fixed high-dose combination and a third controller (tiotropium, antileukotriene, doxifylline). Exclusion criteria included contraindications to clinical exercise tests (Holland et al., 2014), comorbidities affecting exercise capacity, treatment with β-blockers and inability to carry out the study protocol. Subjects were in stable clinical conditions at the time of enrollment. The study protocol was approved by Local Ethics Committee and an informed consent was signed by each subject participating in the study.

### Study design

Clinical assessments, lung function evaluation and 6MWT were carried out on the day of the first administration of Benralizumab (T_0_) and after a period of 6 months (T_1_). Asthma control and quality of life were investigated using the Asthma Control Test (ACT), the Asthma Control Questionnaire (ACQ) and the Asthma Quality of Life Questionnaire (AQLQ).

Forced and slow vital capacity manoeuvres were performed in accordance with ATS/ERS guidelines (Miller et al., 2005; Wanger et al., 2005; Graham et al., 2019). The 6MWT was performed along a flat, straight, 30-m walking course supervised by a well-trained researcher according to ATS guidelines (Holland et al., 2014), using a portable spirometer (Spiropalm; COSMED, Rome, Italy) with integrated pulse oximeter and ventilation (VE) measurement, which allows the measurement of IC at the beginning, at resting conditions (pre-6MWT-IC) and immediately after the end of the test (post-6MWT-IC). DH was defined as a decrease of >150 mL in IC at the end of exercise compared with resting levels (post-6MWT-IC - pre-6MWT-IC) (O’Donnell et al., 2001; Benfante et al., 2018).

### Statistical analysis

The results are expressed as mean and standard deviation, unless otherwise specified. Statistical analysis was conducted via RStudio system. The quantitative variables were analyzed using a paired t-test. P-values <0.05 were considered statistically significant.

## Results

**FIGURE 1:**
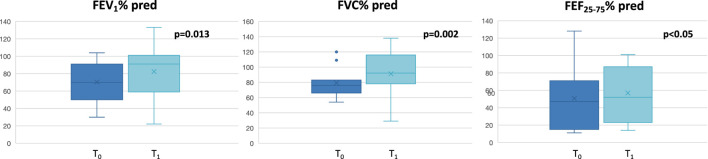
Changes in lung function assessments at T_0_ and at T_1_. FEV_1_: Forced Expiratory Volume in the first second; FVC: forced vital capacity; FEF_25-75_: Forced expiratory flow at 25%–75% of the vital capacity.

**FIGURE 2:**
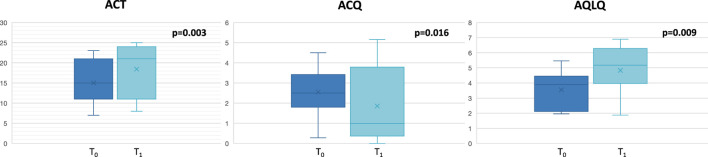
Changes in asthma control and quality of life at T_0_ and at T_1._ ACT: Asthma Control Test; ACQ: Asthma Control Questionnaire; AQLQ: Asthma Quality of Life Questionnaire.

Twelve patients (8 females and 4 males, age:57 ± 8.6 years) affected by severe eosinophilic asthma, meeting the eligibility criteria for benralizumab treatment, were recruited. All severe asthmatics enrolled in the current study were never smokers. The baseline characteristics of the enrolled patients are depicted in Table 1. No exacerbations were documented during the study period. As expected, significant improvement of dynamic volumes (Figure 1), asthma control and quality of life (Figure 2) was observed after anti IL-5R treatment (Table 2).

**TABLE 1:** Demographic and clinical data, biological and lung function evaluations of the asthmatic subjects at baseline.

Characteristic	Total (n=12)
Demographic data	
Female sex (n)	8
Age at study enrolment (years)	57.2 ± 8.63
BMI (kg/m^2^)	28.83 ± 6.19
Clinical data	
Atopic — yes (%)	6 (50)
CRwNP — yes (%)	7 (58.3)
Bronchiectasis — yes (%)	2 (17)
GERD — yes (%)	8 (66.7)
Obesity — yes (%)	3 (25)
Depression/Anxiety — yes (%)	3 (25)
Osteoporosis — yes (%)	3 (25)
Obstructive sleep apnea syndrome — yes (%)	2 (17)
Pharmacologic therapies	
High dose ICS-LABA, n (%)	12 (100)
LAMA, n (%)	7 (58.3)
LTRA, n (%)	7 (58.3)
Previous anti-IgE/anti IL-5 mAbs, n (%)	4 (33.3)
Patients on OCS, n, (%)	5 (42)
OCS, mg/day, mean (SD)	8.5 (6.5)
Biological data	
Eosinophils in blood (cells/mm^3^)	792.6 ± 466.35
Lung function evaluations	
FEV_1_% predicted	70.27 ± 24.76
FVC% predicted	79.45 ± 19.93
FEV_1_/FVC	0.73 ± 0.18
FEF _25-75_% predicted	50.64 ± 35.06
Asthma and quality of life scores	
ACT	15 ± 5.71
ACQ	2.54 ± 1.28
AQLQ	3.55 ± 1.28

Data are presented as mean±SD, unless otherwise stated. ACT: Asthma Control Test; ACQ, Asthma Control Questionnaire; AQLQ, Asthma Quality of Life Questionnaire; BMI, Body Mass Index; FEV_1_, Forced Expiratory Volume in 1 s; FVC, Forced Vital Capacity; FEF_25–75_%, Forced Expiratory Flow at 25–75% of FVC; LABA, Long-Acting Beta agonist; LAMA, Long-Acting Muscarinic Agonist; LTRA, Leukotriene Receptor Antagonist; OCS, Oral Corticosteroids.

**TABLE 2:** Change in clinical and lung function evaluations of the study subjects at T_0_ and T_1_.

Characteristic	T_0_ (baseline)	T_1_ (6 months)	p-value
Lung function evaluations			
FEV_1_% predicted	70.27 ± 24.76	82.45 ± 32.8	0.013
FVC% predicted	79.45 ± 19.93	91.36 ± 30.49	0.002
FEV_1_/FVC	0.73 ± 0.18	0.72 ± 0.12	ns
FEF _25-75_% predicted	50.64 ± 35.06	56.91 ± 32.25	<0.05
Asthma and quality of life scores			
ACT	15 ± 5.71	17.33 ± 7.23	0.003
ACQ	2.54 ± 1.28	1.85 ± 1.89	0.016
AQLQ	3.55 ± 1.28	4.83 ± 1.66	0.009
6-min walk test			
IC pre 6MWT test (L)	2.40 ± 0.48	2.74 ± 1.14	0.010
IC post 6MWT test (L)	1.60 ± 0.83	2.85 ± 1.22	<0.05
VE pre 6MWT test (L·min^−1^)	13.88 ± 4.44	11.35 ± 7.34	<0.05
VE post 6MWT test (L·min^−1^)	23.77 ± 12.11	22.34 ± 7.06	<0.05
6MWD (meters)	393 ± 221	375 ± 186	ns
SpO_2_ pre 6MWT test (%)	95.91 ± 1.08	96.54 ± 1.17	ns
SpO_2_ post 6MWT test (%)	95 ± 2.73	96.36 ± 2.38	ns

Data are presented as mean ±SD, unless otherwise stated. ACT, Asthma Control Test; ACQ, Asthma Control Questionnaire; AQLQ, Asthma Quality of Life Questionnaire; FEV_1_, forced expiratory volume in 1 s; FVC, forced vital capacity; FEF_25–75%_, forced expiratory flow at 25–75% of FVC; IC, inspiratory capacity; SpO_2_, peripheral oxygen saturation; VE, minute ventilation; 6MWD, 6-min walk distance; 6MWT, 6-min walk test.

At T_0_, pre-6MWT-IC and post-6MWT-IC were 2.40 ± 0.48 L and 1.60 ± 0.83 L, respectively (p < 0.0001). Minute ventilation (VE) at the beginning of the 6MWT was 13.88 ± 4.44 L·min^−1^and post 6MWT was 23.77 ± 12.11 L·min^−1^ (p < 0.0001). The distance walked was 393 ± 221 m.

At T_1_, pre-6MWT-IC was 2.74 ± 1.14 L and post-6MWT-IC was 2.85 ± 1.22 L (p = NS); similarly, VE did not change (pre-6MWT vs. post-6MWT: 11.35 ± 7.34 L·min^−1^ vs. 22.34 ± 7.06 L·min^−1^). The average walked distance was 375 ± 186 m).

**FIGURE 3:**
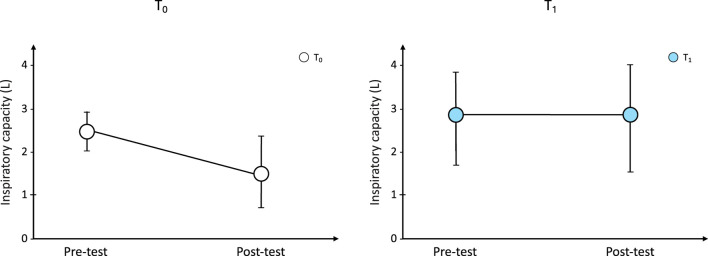
Changes from baseline in IC during the 6-min walk test at T_0_ and after bernalizumab treatment. IC: inspiratory capacity.

When comparing pre-6MWT-IC between T_0_ and T_1_, IC significantly increased after treatment period, indicating the beneficial effect of benralizumab on static hyperinflation (pre-6MWT-IC, T_0_ vs. T_1_: 2.40 ± 0.48 L vs 2.74 ± 1.14 L; p = 0.01). In addition, at T_1_ IC did not change after exercise (p = 0.53) suggesting that DH disappeared after treatment with benralizumab (Figure 3).

No difference in terms of percutaneous oxygen saturation and meters walked in the 6MWT was observed after the treatment period.

## Discussion

DH is considered one of the main factors contributing to asthma symptoms and impaired daily life activity (van der Meer et al., 2019); for this reason, it is candidate to become an important treatable trait in moderate to severe asthma. In this exploratory study, we measured the IC changes to evaluate the occurrence of static and dynamic hyperinflation in experimental settings resembling daily activities. The current findings confirm the occurrence of DH in severe asthmatics, and demonstrate that a 6-month period of treatment with biologic drug is able to 1) significant increase IC at resting condition, suggesting a beneficial action on static hyperinflation, and 2) reduce the occurrence of DH, which disappeared in 10 out of 12 patients.

Static hyperinflation is a functional condition primarily caused by abnormally increased static (or quasi-static) pulmonary compliance, and is expressed by reduced IC. Although this is common in emphysema, there is evidence suggesting that static pulmonary compliance can also increase in severe asthmatics, especially during exacerbation, and return to normal condition after antinflammatory treatment (Gold et al., 1967; Gelb et al., 1998). In subjects with severe airway obstruction and high respiratory rates, with the consequent occurrence of expiratory flow-limitation, DH may occur. It is plausible to conceive that, following pharmacological treatment capable of reducing airway obstruction, IC increases because of lung deflation towards resting conditions of the respiratory system. The current findings support this phenomenon, showing an increase in IC at resting (pre-WT) conditions after treatment with biologic, which paralleled the increase in FEV_1_. As mentioned above, the possibility that the biologic treatment has acted on lung compliance through unknown mechanisms cannot be excluded.

The most interesting observation is that DH disappeared after treatment with benralizumab. A real-life investigation on the effect of benralizumab on lung volumes (Pelaia et al., 2020) demonstrated clinically relevant changes in static lung volumes after 24 weeks of treatment in 22 severe asthmatics. The tendency to reduction in lung hyperinflation was also shown in a larger cohort of severe asthmatics in a phase IIIB study on benralizumab (the SOLANA study) (Panettieri et al., 2020). Maniscalco and coworkers (Maniscalco et al., 2024) conducted a retrospective observational study on the effect of biologics on lung hyperinflation, showing a tendency towards efficacy in reducing lung hyperinflation of biological agents. The novelty of our findings lies in the fact that lung volume changes were assessed with a portable spirometer during the walking test, in a condition that mimics daily common activities. In a previous study, Benfante et al. (2018) highlighted this phenomenon by studying two groups of patients (subject with severe asthma and individuals suffering from COPD) with a similar degree of obstruction. The study showed that severe asthmatics developed dynamic hyperinflation to the same extent of COPD, by means of similar reductions in IC during the WT. Another study showed that patients with history of severe asthma, in particular the “near-fatal” patients, underwent a significant decline of IC during cardiopulmonary exercise test (Rinaldo et al., 2023). These observations clearly confirm that DH occurs in the most severe forms of asthma. Recent observations demonstrated that, unlike to COPD, DH in asthmatics is strictly associated with airway inflammation and can be reduced by systemic anti-inflammatory treatment (van der Meer et al., 2021). In this context, it is possible to consider that a treatment that selectively blocks the inflammatory cascade has beneficial effects on inflamed airways. Taken together, these results suggest that systemic anti-inflammatory treatments, including monoclonal antibodies, may have the potential to positively impact on daily life activities and improve exercise capacity by decreasing DH. Available data support this assumption, by showing an effect of benralizumab on functional indices of peripheral airways (Pelaia et al., 2020; Maniscalco et al., 2024; Chan and Lipworth, 2023).

The increase of IC appears to be associated with important clinical implications in terms of exercise tolerance and perception of dyspnea, more so than the FEV1 parameter. The IC represents an indirect parameter for measuring end-expiratory lung volume, being on the other hand an extremely simple test to perform. It has been shown that the increase in IC, associated with bronchodilator therapy, significantly correlates with dyspnea at rest, with dyspnea during exercise and with physical performance and consequently with quality of life (Pretto et al., 2007). It is known that in asthmatic subjects, poor control of the symptoms of the disease, including dyspnea, is associated with a greater risk of limitation of physical activity, with a negative impact on work and daily life activities (Haselkorn et al., 2010). Carpagnagno et al. investigated the physical activity levels in patients suffering from severe asthma and treated with biologic therapy, and demonstrated that these therapies could be valuable in augmenting the physical activity level in severe asthma (Carpagnano et al., 2019). Lombardi et al. (Lombardi et al., 2023) assessed the clinical response to mepolizumab using the 6MWT, showing the potential of this test to complement the assessment of severe asthma. In this study, 6MWT showed sensitivity to change after asthma treatment and good correlations with asthma symptoms, quality of life and small airway dysfunction.

In conclusion, DH is an important target and an important treatable trait in severe asthma. The current study confirmed that severe asthmatic subjects develop DH during exercise and showed that a 6-month period of treatment with benralizumab in severe eosinophilic asthmatics is able to increase IC significantly at resting condition, suggesting a beneficial action on static hyperinflation, and to reduce significantly the occurrence of DH, impacting on daily activities and quality of life. Further studies are needed to explore these preliminary findings in a larger population of severe asthmatic subjects.

## Data Availability

The raw data supporting the conclusions of this article will be made available by the authors, without undue reservation.
